# The motivation and consequence of fact-checking behavior: An experimental study

**DOI:** 10.1371/journal.pone.0323105

**Published:** 2025-05-23

**Authors:** Valeria Bodishtianu, Dongfang Gaozhao, Pengfei Zhang

**Affiliations:** 1 Department of Economics, Cornell University, Ithaca, New York, United States of America; 2 Department of Political Science, University of Dayton, Dayton, Ohio, United States of America; 3 School of Economic, Political and Policy Sciences, The University of Texas at Dallas, Richardson, Texas, United States of America; Cardiff University, UNITED KINGDOM OF GREAT BRITAIN AND NORTHERN IRELAND

## Abstract

In a series of online experiments, we asked people to evaluate news veracity and varied two experimental conditions: (1) the opportunity to receive fact-checking results and (2) bonus payment for accuracy. We tested three competing theories for fact-checking behavior: value of information (VoI), limited attention (LA), and motivated reasoning (MR). We find that monetary incentives do not promote fact-checking. Prior awareness of the news and perceived easiness in determining news authenticity significantly reduce fact-checking. Democrats are more likely to fact-check on the news aligning with Republicans’ ideology, suggesting a tendency to seek information when there is a need to defend one’s pre-existing belief. Overall, our results contradict VoI, show mixed evidence for MR, and support LA. When available, fact-checking consistently improves subjects’ accuracy in evaluating news veracity by over 40%, underscoring the importance of promoting fact-checking in curbing misinformation.

## Introduction

Online misinformation remains a significant policy concern. At its core, misinformation refers to false information that can be factually falsified and spread regardless of an intent to mislead, which leaves out deceptive disinformation campaigns [1], conspiracy theories, and satire that are challenging to verify [2]. Specifically, misinformation related to politics, public health, climate change, and international conflicts has far-reaching adverse effects on individuals, governments, and society. Misinformation concerning COVID-19 and vaccines, for example, has disrupted the adoption of effective protections against the virus [3]. Tragically, the consequence can be fatal: one estimate suggests that at least 800 people may have died because of misleading and erroneous virus-related information [4]. In an increasingly polarized society with a growing concern about online information being biased and fabricated, creating an environment that systematically fosters critical thinking, reduces misbelief, and cultivates informed citizenship is of great importance.

Previous studies have looked into reasons for individuals to fall into traps of misinformation and strategies to combat misinformation. For example, people may believe false information due to a lack of reasoning [5] or the influence of confirmation bias [6]. Yet little is known about when individuals decide to *actively* seek factual information. Given the great availability of search engines and fact-checking sites, it is a puzzle why people choose not to fact-check and what intervention might incentivize their fact-check behavior.

To answer these questions, this study investigates individuals’ fact-checking decisions in an online controlled experimental setting. We asked our participants to evaluate the veracity of 18 news items that contained true or false information. In a 2×2 between-subject design, we randomly assigned participants to two experimental conditions: (1) the opportunities to receive fact-checking results and (2) bonus payment for accuracy. This experimental design allows us to test three leading explanations for individuals’ information-seeking behavior, namely the value of information (VoI), limited attention (LA), and motivated reasoning (MR).

Our experimental results show that fact-checking significantly increases the accuracy of individuals’ evaluations. The improvement is as high as 40% compared to those who do not receive fact-checking opportunities or bonus payments in the experiment, underscoring the importance of promoting fact-checking in curbing misinformation.

Although fact-checking offers clear benefits, individuals do not always seize the opportunity to fact-check news. In this regard, our results point out several attributes that predict individuals’ fact-checking behavior. Contrary to the VoI predictions, monetary incentives and personal interest do not increase fact-checking. Information incongruence only has a positive impact on Democrats’ fact-checking on news aligned with Republicans’ slant but not vice versa, a piece of mixed evidence for MR. Prior awareness and perceived easiness decrease fact-checking likelihood whereas social importance increases the likelihood. Specifically, in a follow-up experiment, we find that an exogenous increase in prior awareness decreases the likelihood of fact-checking by 15%. These findings are mostly consistent with LA.

This research contributes to the literature by examining the motivation and consequence of fact-checking decisions. Most previous studies on misinformation focused on individuals’ perceptions and evaluations of the news [5, 7–10]. Our paper complements the literature in three aspects. First, our focus shifts from passive exposure to content to an active search for truth. We look into individuals’ active information-seeking behavior after reading the news, which has important implications for evaluating true or false information as a final outcome. Our experimental design allows us to quantify the treatment effects of fact-checking. Second, we distinguish competing theories by deriving testable hypotheses and rigorously test the motivation of fact-checking in a controlled experimental setting. In this way, we identify important mechanisms that can encourage people’s critical thinking and improve their media literacy. Third, our findings shed light on what type of interventions would be effective to nudge fact-checking. For instance, the juxtaposition of opposing views would likely motivate fact-checking for some people, whereas external incentives like cash might not.

## Background and hypotheses

There are three theoretical explanations for individuals’ fact-checking behavior: the value of information (VoI), limited attention (LA), and motivated reasoning (MR). Each of them leads to distinct testable hypotheses of the factors that affect fact-checking decisions.

In VoI, a rational individual would want to seek information only if the additional information improves the decision-making outcome, just like checking the weather would help make a more informed decision about bringing an umbrella. Given that referring to additional information to validate the original information requires time and cognitive effort, only when fact-checking can add value, should individuals engage in such information-seeking behavior. The value of information in our context can be the willingness-to-pay for truth if truth leads to a financially valuable outcome. Prior research has found that individuals are more likely to evaluate information accurately if the accuracy of their evaluation is associated with the amount of monetary incentives they can receive, suggesting the latter’s positive impact on people’s accuracy motivations [11, 12]. We thus expect that providing financial bonuses for being accurate can also increase people’s fact-checking behavior. In this situation, people are expected to fact-check more frequently as a means to reach high accuracy and consequently high bonus. This leads to our first hypothesis.

**Hypothesis 1.** *Monetary incentive increases fact-checking behavior.*

VoI also posits that if a piece of information is personally relevant to people, such as directly involving themselves and speaking to their self-interests, they are more likely to invest in information gathering [13–15]. When people feel interested, the information becomes more valuable to them personally, encouraging systematic processing through greater concerns about forming accurate evaluations [16]. Therefore, we expect that:

**Hypothesis 2.** *Personal interest is positively associated with fact-checking behavior.*

Unlike VoI’s argument that fact-checking is a rational choice, LA views fact-checking as a result of a person’s cognitive constraints. Due to the finite capacity of human attention, people cannot process all relevant information and will thus base their judgments on the limited knowledge they can gather. The discomfort associated with a perceived gap between available and desired knowledge can often lead to increased information-seeking behaviors [17, 18], not only out of a conscious desire to make informed decisions but also due to feelings of anxiety that uncertainty brings [19]. In view of this, prior awareness of the news reduces the inherent feeling of discomfort from the cognitive constraint. As a result, individuals’ confidence in their existing knowledge may lead them to believe they can adequately evaluate the information, reducing their perceived need to fact-check. Based on this, we hypothesize that:

**Hypothesis 3.** *Prior awareness is negatively associated with fact-checking behavior.*

Moreover, as learning becomes increasingly effortful with uncertainty [20, 21], the presence of cognitive constraints can lead individuals to lose motivation to fact-check when they perceive the task of verifying news on their own as straightforward or easy. Put differently, individuals are more likely to use fact-checking when engaging in a piece of news that is challenging to determine its veracity by themselves. In this scenario, people would take the external fact-checking result as a shortcut and rely on its verdict to evaluate information at face value [22]. In this spirit, we form the following hypothesis:

**Hypothesis 4.** *Perceived easiness of determining news veracity is negatively associated with fact-checking behavior.*

Additionally, higher social importance increases the media exposure of the news and is more likely to catch people’s attention for fact-checking [16]. Research has shown that citizens are more willing to seek detailed, relevant information when facing high-salience policy issues such as fracking, compared to low-salience policy issues like storm-water management [23]. This leads to our next hypothesis.

**Hypothesis 5.** *Social importance is positively associated with fact-checking behavior.*

Different from the VoI and LA theories, MR argues that individuals are neither rational nor lazy. Instead, they process information with a directional goal and are motivated to preserve unreasonable expectations and manipulate beliefs in a self-serving way [18, 24, 25]. A good example is voter preferences. When policies align with the political party to which voters belong, those voters may support policies that they would otherwise oppose [26]. This phenomenon is caused by individuals being motivated to arrive at a particular conclusion.

A prominent consequence of motivated reasoning is confirmation bias, which makes people more likely to consciously or unconsciously seek information that affirms their preexisting identities and beliefs [27, 28]. In situations where there is significant uncertainty about how new information — or the results of fact-checking — might align with their current viewpoint, confirmation bias may lead people to avoid fact-checking to preserve their existing beliefs [29–31].

Experimental studies have demonstrated that individuals may perceive the information they favor as truth and unfavorable information as falsehood [11, 32]. This suggests that individuals can be motivated to discredit the validity or quality of messages that do not align with their existing opinions. Such motivations may affect fact-checking behavior, leading people to fact-check messages as a means to invalidate them [9, 33]. Therefore, we predict that:

**Hypothesis 6.** *Processing information congruent with beliefs is negatively associated with fact-checking behavior.*

**Hypothesis 7.** *Processing information incongruent with beliefs is positively associated with fact-checking behavior.*

## Experimental design

To test our hypotheses, we conducted an online experiment on Prolific between October 18-19, 2023, involving 662 participants from the United States. Prolific is a high-quality platform for online experiments that allows researchers to recruit participants from a U.S. representative sample. In our experiment, participants were instructed to judge whether a set of news claims was factually true. The experiment had a 2×2 between-subject design, in which we manipulated two experimental conditions, namely (1) the availability of seeking fact-checking results and (2) the type of monetary incentives respondents would receive, resulting in 4 separate treatment groups. Participants were randomly assigned to one of the treatment groups of which Table 1 provides an overview.

**Table 1:** Experimental conditions.

Treatment group	Fact-checking results	Monetary incentives
T1	Unavailable	Fixed payment
T2	Unavailable	Fixed payment + bonus
T3	Available	Fixed payment
T4	Available	Fixed payment + bonus

In Treatment Group 1 (T1) which serves as a baseline control group, participants were compensated a fixed $3 flat rate for evaluating news items without the option of obtaining fact-checking results. In Treatment Group 3 (T3), participants also received the same fixed payment but had the option to consult fact-checking results. Both Treatment Groups 2 (T2) and 4 (T4) offered participants a $0.20 bonus for each accurate evaluation of news authenticity, defined as correctly assessing whether a given news item was factually true or false, in addition to the fixed $3 payment. The key distinction between T2 and T4 was that participants in T2 did not have access to fact-checking results, whereas those in T4 could click to receive fact-checking results when they deemed it necessary. Although some studies use higher stakes for rewarding accuracy [12, 34], we tested the robustness of our findings by conducting a follow-up experiment featuring a substantially larger bonus payment: $1 for each accurate evaluation, representing a fivefold increase over T4. We found no significant differences across these various bonus scales, and we will discuss these findings in more detail later.

In addition, for participants in T3 and T4 who had access to fact-checking results, they learned from the instructions that they could use up to 9 opportunities to obtain a credible fact-checking result whenever they wanted. Participants in those groups were shown a “Verify the news” button, which showed either a “True” or a “False” message when clicked, truthfully verifying each news item, as long as all 9 attempts had not been used yet. This experimental procedure generated variations in fact-checking decisions at the subject-item pair level, which is crucial in analyzing the causes of fact-checking.

### Procedure

Participants in each treatment group read a total of 18 news items in a random order. For each news item, they must answer 7 related questions designed to assess their perceptions of various aspects of the information, including authenticity, relevance to personal interest, intention to discuss it with family and friends, intention to repost it on social media, easiness of authenticity evaluation, prior awareness, and social importance. The order of the questions was randomized across the participants but kept consistent for each separate run. The participants had a maximum of 2.5 minutes for each item, with a 15-second minimum hold on the page to avoid immediate click-through.

Participants then answered questions about their demographic and socioeconomic information. Upon completion, the experiment debriefed them on information authenticity to avoid possible harm caused by the exposure to misinformation. In the debriefing form, correct answers regarding information authenticity were provided, along with links to news details and explanations for participants’ reference. S1 Appendix shows details of our experimental instructions and procedure.

### News items

We selected 18 news items from the news archive of Snopes, a widely recognized and professional fact-checking source, by which each news item had been rated as either *entirely* “True” or “False.” Snope gives a “True” label if the primary elements of a news claim are demonstrably true and a “False” label if demonstrably false [35]. These ratings are not related to the worthiness of fact-checking. This design choice excludes other categories like “Mostly True,” “Mixture,” and “Mostly False,” reducing ambiguity in news claims and potentially increasing the discernment of true and false content. Each of these news items consisted of a headline, a lead, and an accompanying image related to the news claim. This combination closely mimics the way news is presented on social media platforms like Facebook and X (formerly Twitter). Research has demonstrated that individuals on social media tend to mostly focus on these elements when processing information and getting an update on current affairs [36, 37].

**Fig 1:**
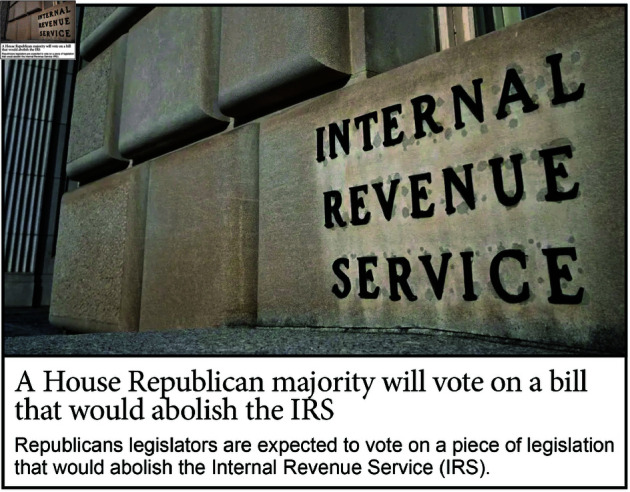
An example of news item.

The news items cover a wide range of political and nonpolitical topics. One-third of these items are either politically neutral or unrelated to politics, such as “Yes, $725M Facebook class action settlement is legitimate.” The remaining two-thirds are divided evenly to align with Democrats or Republicans ideologically. Examples of Democrat- and Republican-consistent news are Roughly 25% of the nation’s debt was incurred during the Trump Administration” and “U.S. President Joe Biden’s administration is planning to ban gas stoves over concerns surrounding climate change,” respectively. Fig 1 demonstrates how a news item was presented and S2 Appendix provides a full list of news items used in the experiment.

### Experiment background

The experiment was created on oTree, an open-source online platform for experiments [38], coded mostly in Python, with some small exceptions for JavaScript when necessary. The application was hosted on the Heroku cloud platform and the link to it was provided in the Prolific survey along with a short description of the experiment. Detailed instructions were provided after the participant clicked on the link and agreed to the consent form. One of the restrictions for the participants was a requirement for taking the survey on a desktop device, to eliminate any possible issues with mobile devices. In addition, participants had to be from the United States and be fluent in English.

## Results

Our analysis begins with a balance test (see S3 Appendix for details). 52.42% of research participants were male and 56.84% of respondents were between the ages of 35 and 64. The sample was disproportionally Caucasians (70.20%) in terms of race and ethnicity. 53.18% of the participants identified themselves as Democrats, while Republicans and Independents were 18.14% and 28.68% respectively. 70.22% of the subjects identified themselves as at least somewhat liberal in terms of ideology. Regarding education and income, 53.31% had a bachelor’s degree or higher, and 59.36% earned a household income of USD 50,000 or greater in the previous year. 82.20% of the subjects followed the current government and public affairs at least some of the time, whereas 72.05% of the subjects reported fact-checking news at least some of the time.

We then compare participants’ characteristics across treatment groups using chi-squared tests. We also conduct pair-wise t-tests on variables such as age, education, and income, and the results are the same with no significant differences across treatment groups. Across the four groups, there are no statistically significant differences in demographics including gender, age, race, ideology, party affiliation, religion, education, and income. Prior exposure to current affairs and fact-checking habits are also balanced across the four groups. The balance test results provide strong support for random assignments in our experiment, allowing us to use T1 as a counterfactual and identify causal effects.

**Table 2:** Summary statistics by treatment group.

	T1	T2	T3	T4	Total
Correctness	0.536	0.542	0.721	0.725	0.632
	(0.163)	(0.157)	(0.145)	(0.143)	(0.177)
Fact check			0.401	0.388	
			(0.153)	(0.152)	
Personal interest	2.116	2.083	2.051	2.091	2.085
	(0.546)	(0.605)	(0.607)	(0.568)	(0.581)
Discuss with family/friends	1.971	1.986	1.899	1.954	1.952
	(0.568)	(0.624)	(0.597)	(0.606)	(0.599)
Repost on social media	1.654	1.639	1.593	1.657	1.636
	(0.545)	(0.610)	(0.596)	(0.581)	(0.583)
Social importance	2.294	2.314	2.269	2.340	2.304
	(0.529)	(0.548)	(0.558)	(0.528)	(0.541)
Perceived easiness	2.728	2.713	2.725	2.658	2.706
	(0.620)	(0.629)	(0.637)	(0.622)	(0.626)
Prior awareness	0.247	0.254	0.250	0.211	0.240
	(0.176)	(0.203)	(0.236)	(0.187)	(0.202)

Means are presented with standard deviations in parentheses.

### Treatments’ effects on accuracy

**Fig 2:**
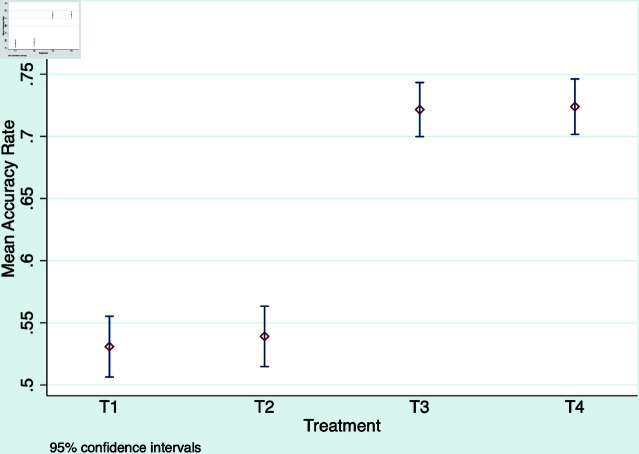
Mean accuracy rate by treatment group.

We then report the average treatment effects of fact-checking and monetary incentives on the accuracy rate. Fig 2 plots the 95% confidence interval of the mean accuracy rate aggregated over all of the rounds for each treatment. Our subjects’ baseline knowledge of the selected news is slightly above a coin flip (56%), providing much room for the role of fact-checking. As shown in the graph, the mean accuracy rate in T1 is significantly greater than 50% (p=0.03, signed rank test). As a sanity check, each subject has 9 opportunities for fact-checking in T3 and T4, so in these two treatments, the accuracy rate is expected to be above 50%. This is confirmed by a one-sided signed rank test. Paying a bonus for correctness, however, does not improve the baseline accuracy rate further. The mean accuracy rate in T2 is 0.6% higher than that in T1, but the difference is not statistically significant. Likewise, the mean accuracy rate in T3 is not statistically different from that in T4. Opportunities for fact-checking, however, do improve the accuracy rate substantially. The mean accuracy rate in T3 is 18.5% higher than that in T1, and the difference is statistically significant. Similarly, the mean accuracy rate in T4 is statistically higher than that in T2. Yet, fact-checking does not eliminate misperception. The mean accuracy rates in T3 and T4 are significantly lower than 100% (*p* = 0.008, signed rank test).

**Fig 3:**
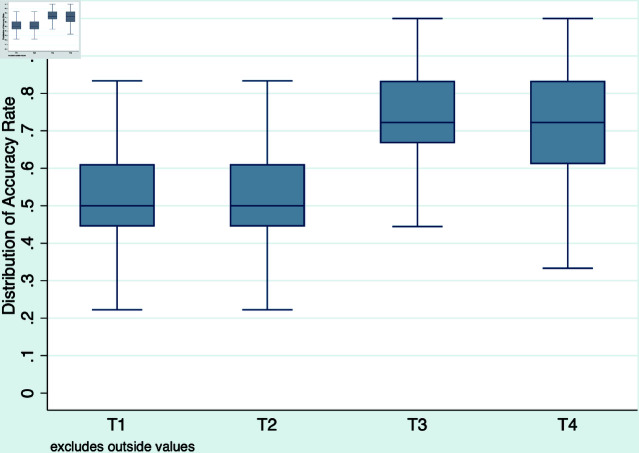
Box plots of individual accuracy rate by treatment group.

Fact-checking not only improves the accuracy of an average subject but also shifts up the entire distribution. Fig 3 shows the distribution of individual accuracy rates by quartiles for each treatment. The opportunities for fact-checking lead to a significant increase in accuracy rate for all the quartiles. In T3 and T4, some subjects correctly assess all the news items, whereas there are no such subjects in T1 and T2. The non-parametric Mann-Whitney test confirms that the distribution of accuracy rates in T3 is a significantly upward shift from that in T1 (*p* = 0.008). Likewise, the distribution of accuracy rates in T4 is significantly different from that in T2 (*p* = 0.02). We also observe that treatment groups that receive bonuses have a very similar accuracy distribution as treatment groups getting a fixed rate. The Mann-Whitney test confirmed that the distribution of accuracy rates in T2 is not significantly different from that in T1 (*p* = 0.62). The same comparison holds for T4 and T3 (*p* = 0.38).

**Result 1.** *In all of the treatment groups, the mean accuracy rates are significantly greater than 50%, and significantly less than 100%.*

**Result 2.** *Monetary incentives do not improve the accuracy of an average subject evaluating news authenticity.*

Our experimental design allows us to causally estimate the treatment effects of fact-checking and bonus payment on accuracy. We estimate the following two-way fixed-effect (TWFE) model at the level of individual-item pairs:

$$Correct_{it}=\alpha_{i}+\eta_{t}+\beta \cdot Treatment_{i}+\varepsilon_{it}$$ (1)

where *i* is a subject, *t* is an item, *Correct*_*it*_ is an indicator variable that takes the value of1 if subject *i* is correct about the authenticity of item *t*, and takes the value of 0 otherwise. *Treatment*_*i*_ is an indicator variable for whether subjecti is in the treatment group *Treatment*_*i*_ = 1 or the control group *Treatment*_*i*_ = 0. $\alpha_{i}$ is the individual fixed effect, and $\eta_{t}$ is the item fixed effect. The standard errors are clustered at the subject level. The coefficient β identifies the average treatment effect of fact-checking and bonus payment on accuracy.

Table 3 reports the estimated effect of fact-checking on the accuracy rate. The full analysis of secondary determinants of accuracy, based on participants’ assessment of news attributes, is delegated to the S4 Appendix. Six specifications are reported, where the set of covariates is sequentially expanded. The regression results suggest a significant positive relationship between fact-checking and accuracy. Holding all other things constant, fact-checking increases the accuracy rate by 40%. The estimated effect is robust to the inclusion of item fixed effect or the two-way fixed effects. To put it in context, the effect on accuracy is more than 2 standard deviations above the mean.

**Table 3:** The effect of fact-checking on individual accuracy rate.

	(1)	(2)	(3)	(4)	(5)	(6)
	**LPM**	**LPM**	**LPM**	**LPM**	**Logit**	**Probit**
Fact-check treatment	0.391^***^	0.391^***^	0.408^***^	0.401^***^	3.662^***^	2.038^***^
	(0.0107)	(0.0107)	(0.0103)	(0.0105)	(0.131)	(0.0662)
Bonus treatment		0.00938	0.00961	0.0113	0.351	0.185
		(0.0104)	(0.00991)	(0.125)	(0.910)	(0.540)
Item fixed effect	No	No	Yes	Yes	Yes	Yes
Individual fixed effect	No	No	No	Yes	Yes	Yes
Observations	6048	6048	6048	6048	5904	5904
Adjusted *R*^2^	0.182	0.182	0.260	0.299		
Standard errors in parentheses. ^*^ *p*<0.05, ^* *^ *p*<0.01, ^* * *^ *p*<0.001						

**Result 3.** *Opportunities to receive fact-checking results increase the accuracy of an average subject evaluating news authenticity by 40%.*

Notably, a previous study also shows fact-checking improves factual knowledge regarding policy issues in an experimental setting [39]. Our paper is complementary to it in three aspects: (1) Their design focuses on the debate on immigration policy in the 2017 French presidential election, while ours covers more issues in the U.S. including national debt, environmental regulation, social media, etc. (2) They are interested in the effects of fact-checking results but not the fact-checking behavior per se. Their design does not enable the participants to actively seek information, i.e., all subjects in their fact-checking treatment are presented with the official statistics. (3) They extensively measure voting intentions, which goes beyond the scope of this paper.

### What motivates fact-checking

**Fig 4:**
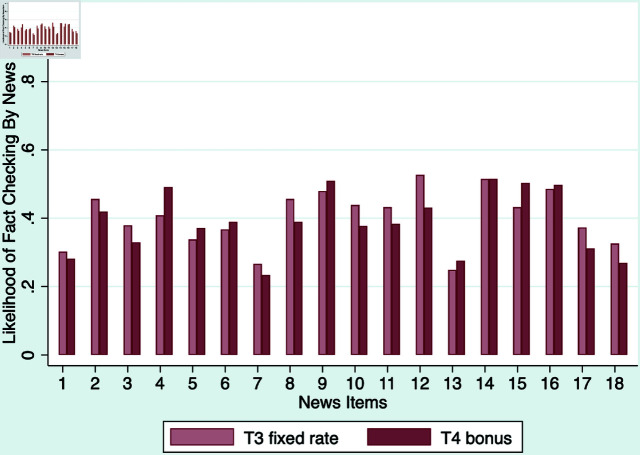
Histogram of fact-checking likelihood by news items.

We now shift gear to the determinants of subjects’ fact-checking behavior. Recall that only participants in T3 and T4 had the opportunity to access fact-checking results. Importantly, we observe that the manner in which participants were incentivized monetarily does not significantly influence their inclination to fact-check news items, although the fact-checking likelihood varies considerably across news items, as demonstrated in Fig 4 and S5 Appendix.

**Result 4.** *Monetary incentives do not increase fact-checking behavior, which contradicts Hypothesis 1.*

For this reason, we follow up by examining the relationship between participants’ fact-checking behavior and their assessment of the news’ attributes. Our following discussion is based on binary regressions in which we regress participants’ binary fact-checking decisions on their 7-dimension assessments of the attributes, with the item and individual fixed effects. Note that the results are robust if we use the linear probability model, logit model, and probit model as alternatives. Formally, we estimate the following TWFE model at the level of individual-item pairs:

$${\text{Fact-check}}_{it}=\alpha_{i}+\eta_{t}+{𝐗}_{it}\cdot \gamma +\varepsilon_{it}$$ (2)

where *i* is a subject, *t* is an item, $\alpha_{i}$ is the individual fixed effect, and $\eta_{t}$ is the item fixed effect. ${\text{Fact-check}}_{it}$ is an indicator variable that takes the value of 1 if subject *i* chooses to fact-check the authenticity of item *t*, and takes the value of 0 otherwise. ${𝐗}_{it}$ is a vector of factors that vary across the individual-item pairs. The standard errors are clustered at the individual level.

The coefficient γ measures the effects of different factors on the likelihood of fact-checking. The TWFE model allows us to rule out various concerns that could otherwise lead to a confounding relationship. First, we can rule out that the results are driven by individual characteristics, including age, race, gender, ideology, etc. We also decompose the individual fixed effect to test the effects of various demographic factors on fact-checking. Second, we can rule out that the results are driven by variations in item types, including true or false, topics, length, slants, etc.

**Fig 5:**
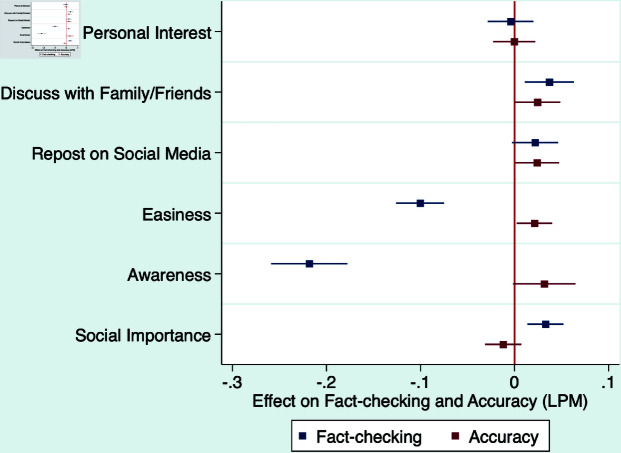
Likelihood of fact-checking versus being accurate.

**Fig 6:**
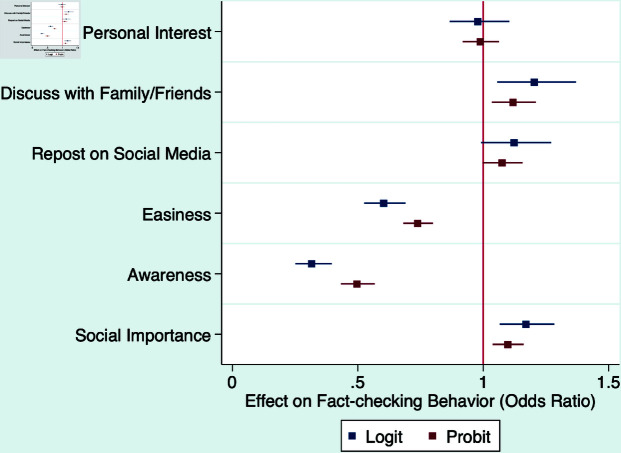
Likelihood of fact-checking a news item (logit and probit).

**Fig 7:**
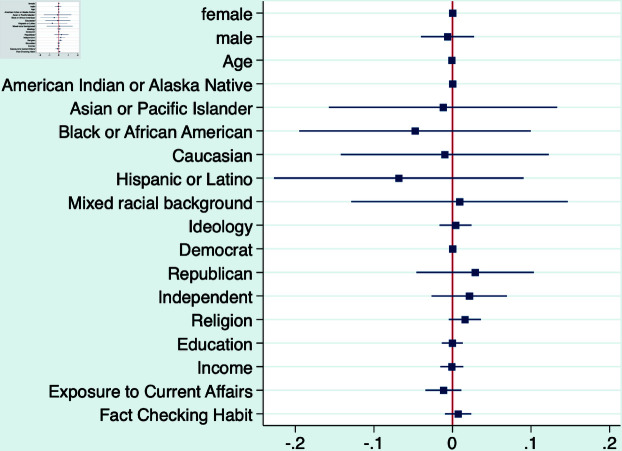
Effects of personal characteristics on fact-checking behavior (LPM).

Table 4 reports the results in different models and Figs 5, 6, and 7 visualize these results. Beginning with respondents’ perceptions of personal relevance, our findings indicate that their intention to engage in discussions about the news with family or friends has a notable and positive effect on their propensity to seek out and consult fact-checking results. However, it is noteworthy that the degree of relevance to respondents’ personal interests or their desire to share the information on social media does not significantly affect their likelihood of engaging in fact-checking.

**Table 4:** Determinants of fact-checking.

	(1)	(2)	(3)	(4)	(5)
	**LPM**	**LPM**	**LPM**	**Logit**	**Probit**
Personal interest	−0.00687	−0.00393	−0.00423	−0.0225	−0.0125
	(0.0107)	(0.0107)	(0.0124)	(0.0618)	(0.0376)
Discuss with family/friends	0.0135	0.0181	0.0370^**^	0.185^**^	0.112^**^
	(0.0122)	(0.0124)	(0.0132)	(0.0664)	(0.0400)
Repost on social media	−0.00510	−0.00783	0.0217	0.115	0.0715
	(0.0116)	(0.0116)	(0.0125)	(0.0634)	(0.0378)
Perceived easiness	$-0.0529^{***}$	$-0.0475^{***}$	$-0.100^{***}$	$-0.507^{***}$	$-0.304^{***}$
	(0.0112)	(0.0111)	(0.0130)	(0.0697)	(0.0410)
Prior awareness	$-0.217^{***}$	$-0.195^{***}$	$-0.218^{***}$	$-1.155^{***}$	$-0.702^{***}$
	(0.0185)	(0.0191)	(0.0206)	(0.117)	(0.0691)
Social importance	0.0499^***^	0.0423^***^	0.0328^***^	0.156^***^	0.0930^***^
	(0.00913)	(0.00983)	(0.00976)	(0.0474)	(0.0289)
Item fixed effect	No	Yes	Yes	Yes	Yes
Individual fixed effect	No	No	Yes	Yes	Yes
Observations	6048	6048	6048	5688	5688
Adjusted *R*^2^	0.054	0.064	0.127		
Standard errors in parentheses. ^*^ *p*<0.05, ^* *^ *p*<0.01, ^* * *^ *p*<0.001						

**Result 5.** *Personal interest is not positively associated with fact-checking behavior to a statistically significant level, contradicting Hypothesis 2.*

**Result 6.** *Intention to discuss with family or friends about the information is positively related to fact-checking behavior.*

Next, we discuss the effects of respondents’ perceived easiness in determining the authenticity, their prior awareness, and perceived social importance of the news. Fig 5 compares the effect of different factors on fact-checking with their direct effect on accuracy. In T3 and T4, the effect of a factor on accuracy would be the combination of the direct effect of the factor and the indirect effect of the factor through fact-checking. We use T1 and T2 as counterfactuals to estimate the direct effect on accuracy. We observe that both perceived easiness and prior awareness have a minimal direct effect on accuracy, however, they significantly reduce the likelihood of fact-checking, making the combined effect on accuracy largely negative. This suggests that our subjects might overly estimate the accuracy of their own judgment, which deters their fact-checking behavior. In addition, subjects who place higher social importance on the news are more motivated to fact-check the specific items.

**Result 7.** *Prior awareness is negatively related to fact-checking behavior, which supports Hypothesis 3.*

**Result 8.** *Perceived easiness is negatively associated with fact-checking behavior, which supports Hypothesis 4.*

**Result 9.** *Social importance is positively associated with fact-checking behavior, which supports Hypothesis 5.*

Importantly, we do not discern any significant effect of respondents’ personal characteristics, such as demographics and socioeconomic status, on their decisions regarding fact-checking. Moreover, the nature of information authenticity, whether the news item is true or false, does not exert a significant effect on individuals’ fact-checking behavior either. In other words, people do not display a greater or lesser aptitude for seeking additional information when confronted with either accurate or inaccurate information.

**Table 5:** Effects of information (in)congruence on fact-checking behavior.

	(1)	(2)	(3)	(4)	(5)
	**LPM**	**LPM**	**LPM**	**Logit**	**Probit**
Democrat slant	0.0334*	0.0798	0.232*	1.000*	0.610*
	(0.0159)	(0.0706)	(0.108)	(0.467)	(0.287)
Republican slant	0.0169	0.0171	0.0573	0.273	0.159
	(0.0150)	(0.0551)	(0.105)	(0.452)	(0.278)
Democratic Party		−0.0445	−0.0533	−0.237	−0.145
		(0.0293)	(0.0327)	(0.141)	(0.0869)
Republican Party		0.0474	−0.0241	−0.104	−0.0652
		(0.0424)	(0.0521)	(0.216)	(0.134)
Democrat slant × Democratic Party		0.0452	0.0439	0.196	0.120
		(0.0358)	(0.0401)	(0.170)	(0.105)
Democrat slant × Republican Party		−0.0169	0.0301	0.129	0.0825
		(0.0534)	(0.0684)	(0.284)	(0.176)
Republican slant × Democratic Party		0.0709*	0.0927*	0.405*	0.249*
		(0.0342)	(0.0383)	(0.165)	(0.101)
Republican slant × Republican Party		−0.0250	0.0329	0.144	0.0899
		(0.0499)	(0.0674)	(0.279)	(0.173)
News literacy controls	No	Yes	Yes	Yes	Yes
Demographics controls	No	No	Yes	Yes	Yes
Observations	6048	5868	5580	5580	5580
Standard errors in parentheses. ^*^ *p*<0.05, ^* *^ *p*<0.01, ^* * *^ *p*<0.001						

Turning to information congruence, we introduce an interaction term between information slant (i.e., Democrat-consistent, Republican-consistent, and politically neutral news) and participants’ party identity. This extended model allows us to examine how individuals fact-check when processing information that aligns or conflicts with their preexisting beliefs. Our regression results show that Democrat-consistent news is more likely to undergo fact-checking, while Republican-consistent news is not. Moreover, neither Democrats nor Republicans display a higher propensity to engage in fact-checking. However, Republican-consistent news is more likely to be fact-checked by Democrat respondents, whereas no statistically significant relationships were observed for other combinations. This effect of confirmation bias is robust to alternative model specification, and the inclusion of news literacy and demographics controls. Therefore, we find partial support for Hypothesis 7 and must reject Hypothesis 6 related to information congruence. This suggests a pattern where individuals from one party may be more inclined to scrutinize information that aligns with the opposing party’s stance, possibly driven by a desire to verify or challenge the claims made by the opposing side. It is also noteworthy that such willingness appears to be asymmetric, with Democrat respondents showing a greater propensity to fact-check Republican-consistent news than vice versa. S6 Appendix shows the robustness of the motivated reasoning results in alternative classification of news items and alternative indicators of information incongruence.

**Result 10.** *Information congruence does not affect fact-checking behavior, which contradicts Hypothesis 6.*

**Result 11.** *Information incongruence is positively associated with fact-checking behavior among Democrats but not Republicans, which partially supports Hypothesis 7.*

The regression results of the factor models can be best interpreted as how well different factors *predict* fact-checking behavior. In doing so, we identify interesting relationships between self-reported item attributes and fact-check propensity and examine whether the TWFE results are consistent with the correlational claims in Hypotheses 2-7. So far, the 7 dimensions of the attributes are rated by the subjects instead of being experimentally manipulated. Experimental manipulation of situational features in fact-checking is always challenging. See this study [40] for some very interesting efforts in this direction, in which the authors randomly assign the subjects to different priming messages, including social pressure, civic duty, patriotism, etc. In what follows, we provide causal evidence for the effects of limited attention on fact-checking behavior by experimentally varying the degree of prior awareness in a follow-up experiment.

### Further experimental evidence for limited attention

To further substantiate the regression findings, we provide causal evidence for the theory of limited attention. In particular, we test Hypothesis 3 in a controlled experimental setting by conducting a follow-up experiment on T3, which we refer to as treatment group T3F. The follow-up experiment was administered on Prolific between February 18 and March 5, 2025. 111 subjects, who were in the original T3, completed T3F, resulting in a 66% response rate. T3F is similar to T3 with the same procedure, the same amount of fixed payment ($3), and the same number of opportunities to receive fact-checking results (9 times). The only difference between T3F and T3 is the prior exposure to some news items that have been experimentally manipulated.

In the follow-up experiment, participants were invited back to evaluate a combination of previous and new items. We randomly selected 9 news items from T3 and repeated them in T3F. A before-after comparison of the fact-checking behavior on the 9 repeated items is a more credible estimate of the treatment effect of prior awareness than the correlational evidence of Result 7. Meanwhile, to have a baseline, we randomly selected another 9 news items from Snopes that had not been previously shown to the participants (see S2 Appendix for these items) and used their decisions on the non-repeated items to measure any unobserved changes.

In doing so, we have a difference-in-difference (DID) design that allows us to causally estimate the treatment effect of prior awareness on fact-checking. Unlike other DID design using observational data where a treatment might not be randomly assigned [41], the treatment of whether an item is repeated or not in T3F is randomly assigned to the news items, thereby avoiding the selection into treatment problem. We estimate the following model at the level of individual-item pairs:

$${\text{Fact-check}}_{it}=\beta_{0}+\beta_{1}{\text{Post}}_{i}+\beta_{2}{\text{RepeatedItem}}_{t}+\beta_{3}{\text{Post}}_{i}\cdot {\text{RepeatedItem}}_{t}+\varepsilon_{it}$$ (3)

where the new variable ${\text{Post}}_{i}$ is a time indicator that takes the value of 1 if it is the post-awareness group T3F and 0 if it is the pre-awareness group T3. ${\text{RepeatedItem}}_{t}$ is an indicator variable that takes the value of 1 if item *t* is a repeated item in both T3 and T3F, and 0 otherwise. The standard errors are clustered at the subject level. The coefficient on the interaction term $\beta_{3}$ is the coefficient of interest that identifies the average treatment effect of prior awareness.

Table 6 reports the DID estimate of the effect of increased prior awareness on fact-checking likelihood. The baseline model in column (1) shows that exogenous changes to prior awareness decrease the likelihood of fact-checking by 15%. The estimated effect is robust to different specifications including the logit and the probit models. The results are consistent with Result 7 and support the theory of limited attention.

**Table 6:** DID estimates of the effect of prior awareness on fact-checking.

	(1)	(2)	(3)
	**LPM**	**Logit**	**Probit**
Post treatment	0.0384	0.159	0.0991
	(0.0215)	(0.0895)	(0.0556)
Repeated item	0.0291	0.121	0.0752
	(0.0199)	(0.0829)	(0.0515)
Post × Repeated	$-0.148^{***}$	$-0.636^{***}$	$-0.392^{***}$
	(0.0405)	(0.182)	(0.111)
Observations	3130	3130	3130
Adjusted *R*^2^	0.003		
Standard errors in parentheses. ^*^ *p*<0.05, ^* *^ *p*<0.01, ^* * *^ *p*<0.001						

**Result 12.** *Prior awareness of news items leads to lower fact-checking likelihood, providing causal evidence for Hypothesis 3.*

## Discussion and conclusion

In this study, we asked when individuals would fact-check information. Before going further into a discussion of our findings, several limitations of this study should be noted. First, our findings are based on concise fact-checking results that only show “True” or “False” messages without any detailed explanation of information authenticity. While we chose this design to avoid variances among different news items, we acknowledge that people in reality may receive more information than this simple message when they fact-check things. Those pieces of contextual information may add or reduce cognitive difficulties in gauging information authenticity, consequently encouraging or discouraging people’s fact-checking behavior.

Second, we recognize that to some readers, our initial bonus payment may be perceived as a low-stake incentive, although such payment is common in online experiments to incentivize subjects’ efforts [42, 43]. To address this concern, we conducted a follow-up experiment specifically testing whether a larger incentive would have a different effect on subjects’ fact-checking behavior. We increased the bonus payment to $1 per correct response, a fivefold increase from the original amount that aligns our design with some of the higher-paying studies in the literature (e.g., [12, 34]). However, we found no significant increase in fact-checking rates or accuracy, consistent with our main findings (see S7 Appendix for details). The ineffectiveness of monetary incentives despite substantial increases in the reward suggests that there are significant mental or psychological barriers to improving fact-checking rates and reducing misinformation beliefs.

Through an experimental design, we find that fact-checking improves the accuracy of evaluating news veracity substantially. The immediate question is how we can promote fact-checking behavior. We present three competing theories on why people (do not) fact-check. Our experimental results do not support the value of information theory. Contrary to VoI, we find that neither monetary incentive nor personal interest increases fact-checking behavior.

Instead, our empirical findings are consistent with certain aspects of the theory of motivated reasoning. As predicted by this theory, information incongruent with prior beliefs increases fact-checking. This is evident in the case of Democrat participants in our experiment, who exhibit a propensity to verify information that aligns with Republican viewpoints. This reckons the survey findings that Democrats show lower trust in the other party [44], and consequently are motivated to fact-check these pieces of information as a way to challenge their authenticity. Moreover, our findings contribute additional nuance to the work of Walter *et al*. [18], who report null effects of ideological (in)congruence on individuals’ propensity to fact-check. Through subgroup analyses stratified by political affiliation, we observe discernible differences between Democrats and Republicans in their likelihood to engage in fact-checking, suggesting that ideological orientation may exert heterogeneous effects that are obscured when examined at the aggregate level. This disaggregation offers a plausible explanation for the null findings in previous research and underscores the importance of accounting for subgroup variability in studies of political cognition and information verification behavior. Furthermore, while Walter *et al*. [18] demonstrate that, when presented with a single news article concerning gun policy, individuals are more inclined to fact-check the information that they perceive as accurate, as a means of confirming their preexisting beliefs, our study encompasses a broader array of topics with varying degrees of social salience and tests multiple theories including motivated reasoning. The divergent findings between the two studies suggest that fact-checking behavior may exhibit issue-specific dynamics and call for further research.

Why do people not fact-check enough? Our results support the theory of limited attention. Perceived easiness and prior awareness decrease fact-checking likelihood. The social importance of the news catches people’s attention and encourages fact-checking in light of their limited attention in the online environment. On the other hand, feeling easy about determining news veracity and having prior knowledge about the news discourages fact-checking behavior. This is in line with previous research [45], which shows that prior exposure can subsequently lead to an increased false perception of accuracy for misinformation. This tendency to rely on preexisting beliefs and information without subjecting them to scrutiny creates a fertile ground for the spread and acceptance of false information.

Our findings also dig into the relationship between fact-checking and social media sharing. Individuals are more inclined to verify the authenticity of information when they intend to discuss it within their social networks, possibly driven by concerns related to reputation management. When juxtaposing this result with people’s intention to repost information on social media, it is likely that individuals’ close social ties have higher levels of interpersonal closeness, trigger a motive to protect others, and intensify the negative consequences of spreading inaccurate information [46, 47]. This, in turn, encourages individuals to place greater emphasis on ensuring the authenticity of the information they share.

Our findings give us some clues on policy options to combat misinformation. Limited attention prevents individuals from conducting critical examinations on every piece of news they receive. As our world is increasingly overloading with information, people do not have the luxury to fact-check every information seriously and are forced to prioritize certain information deemed worthy of their attention [5]. Social media users are more likely to share false news when they find it more novel and emotional [10]. Our findings add that once they share, more users will be exposed to the false news, leading to an increase in prior awareness and consequently a decrease in the fact-checking rate. Thus, the “first few seeds” are critical in determining whether herding of misinformation may happen. Meanwhile, motivated reasoning, including confirmation bias and overconfidence, is a significant behavioral obstacle to fact-checking. This is especially concerning in a society with a growing polarization of wealth and ideology, where many narratives can easily be undermined and disregarded with a simple rallying cry of “fake news.” And money, as shown in our study, does not solve the problem. All of these phenomena call for intervention either by the platforms or by the governments.

In this regard, content moderation, a governance mechanism adopted by social media platforms that removes inappropriate and misleading content, might be a promising approach to regulating the flow of information online [48]. There are a variety of methods to moderate misinformation [49]. One simple way is flagging. Flagging information falsehood has been found useful, substantially shaping how people perceive information authenticity [7]. Our findings suggest that such interventions would be more effective if they are targeted interventions on the “first few seeds” of any misinformation cascades. The optimal intervention, which might crowd in individual fact-checking and prevent herding, remains a promising topic for future research.

## Supporting information

S1 Appendix Experimental instructions. Contains the instructions provided to participants prior to the experiment
(PDF)

S2 Appendix News items used in the experiment. Lists all news items used in the experiments, categorized as false or authentic.
(PDF)

S3 Appendix Balance test. Presents a table of individual characteristics by treatment group.
(PDF)

S4 Appendix Factor model of accuracy rate. Describes a two-way fixed-effects regression analysis of the relationship between accuracy and item-level evaluations.
(PDF)

S5 Appendix Item-wise comparison of fact-checking likelihood. Reports item-level average fact-checking rates across treatment groups.
(PDF)

S6 Appendix Alternative classification of news items. Re-evaluates the main results using an alternative classification of news items.
(PDF)

S7 Appendix Additional treatment of large bonus. Reports results from a follow-up experiment with a significantly larger bonus for each accurate judgment.
(PDF)
