# Supplementary material for: The motivation and consequence of fact-checking behavior: An experimental study
Source: PLoS One. 2025 May 23;20(5):e0323105. doi: 10.1371/journal.pone.0323105 (PMC12101777; doi:10.1371/journal.pone.0323105)
Supplement: S1 Appendix — Contains the instructions provided to participants prior to the experiment (PDF) [file pone.0323105.s001.pdf]

S1 Appendix. Experimental instructions.

You will be presented with 18 news headlines and asked to evaluate them. The primary question will be whether or not you believe the news article is truthful. It is important to use your best judgment [*for participants in T2 and T4 that earn a bonus:* because you will receive a $0.2 bonus for each accurate evaluation of news authenticity (on top of your participation fee).].

[*For participants in T3 and T4 that have access to fact-checking results:* For a total of 18 news headlines, you have 9 opportunities to verify the accuracy of the news. These verifications provide you with credible information about the authenticity of the news. You can choose to use these hints whenever you want.]

On the next page, please read the news headlines carefully and choose the answer that best describes your beliefs. For each news headline, you will have 150 seconds to answer 7 questions.

[*For each news item, participants answer 7 questions related to the information:*]

1. According to your best judgment, is this news claim authentic?

   [1] Yes [0] No

   [*For participants in T3 and T4 that have access to fact-checking results:* A button of "Show fact-check result"]

   [*On the next page, Questions 2 to 6 were asked and randomized between participants but following the same order within participants.*]

2. To what extent do you agree this news is related to your interest?

   [1] Strongly Disagree [2] Disagree [3] Agree [4] Strongly Agree

3. To what extent do you agree you would discuss this news with your family or friends?

   [1] Strongly Disagree [2] Disagree [3] Agree [4] Strongly Agree

4. To what extent do you agree you would repost this news on social media?

   [1] Strongly Disagree [2] Disagree [3] Agree [4] Strongly Agree

5. To what extent do you agree this news is important to society?

   [1] Strongly Disagree [2] Disagree [3] Agree [4] Strongly Agree

6. To what extent do you agree it is easy to determine the authenticity of this news?

   [1] Strongly Disagree [2] Disagree [3] Agree [4] Strongly Agree

7. Were you aware of this news (e.g., reading relevant news before or having an impression of it) before this study?

   [1] Yes [0] No
