# Supplementary material for: The motivation and consequence of fact-checking behavior: An experimental study
Source: PLoS One. 2025 May 23;20(5):e0323105. doi: 10.1371/journal.pone.0323105 (PMC12101777; doi:10.1371/journal.pone.0323105)
Supplement: S2 Appendix — Lists all news items used in the experiments, categorized as false or authentic. (PDF) [file pone.0323105.s002.pdf]

## S2 Appendix. News items used in the experiment.

False news claims

1 Rihanna wore a “satanic” pentagram symbol on her costume while performing at the Super Bowl. Rihanna wore a “satanic” pentagram symbol on her costume while performing at the Super Bowl LVII halftime show on Feb. 12, 2023.

2 Federal law does not give U.S. vice presidents authority to declassify government documents. The discussion surfaced after news broke that two batches of classified documents were found at Joe Biden’s home and former office.

3 Biden’s administration is planning to ban gas stoves over concerns surrounding climate change. Harmful pollutants that cause a number of ailments are reportedly being released by the appliances.

4 The “AR” in AR-15 stands for assault rifle. The weapon was developed in the 1950s with the original intention that it would be used by the military.

5 Fox News changed its accreditation from “news” to ”entertainment.” Fox News has changed its accreditation and now is in the same category as Saturday Night Live, Laugh-In, and Swamp People.

6 Elon Musk’s jet was flown 12 times to convicted sex offender Jeffrey Epstein’s island. An image shows that, since 2010, Elon Musk’s jet was flown 12 times to convicted sex offender Jeffrey Epstein’s island, Little Saint James.

7 Congress officially banned TikTok. The video platform TikTok had been banned from general use in the U.S. after TikTok CEO Shou Zi Chew testified before the U.S. Congress.

8 Elon Musk deleted a Twitter thread on ‘how safe Tesla cars are.’ On Jan. 7, 2023, Elon Musk deleted a Twitter thread from his personal account about how safe Tesla cars are.

9 Facebook rule allows the company to use your photos in court and also makes your posts public. A new Facebook rule will allow the company to use your photos in court and also makes all of your posts and deleted messages public.

True news claims

10 Republican Party in Lee County passed a proposal to ban the sale of COVID-19 vaccines in Florida. The Lee County (Florida) Republican Party claimed to be acting on “behalf of the preservation of the human race.”

11 Some GOP members of Congress were photographed wearing AR-15-shaped pins in February. At least two members of Congress wore the lapel pins, and a third had taken credit for their distribution.

12 A House Republican majority will vote on a bill that would abolish the IRS. Republicans legislators are expected to vote on a piece of legislation that would abolish the Internal Revenue Service (IRS).

13 ChatGPT declined to write a poem admiring Trump, but successfully drafted one for Biden. The artificial intelligence tool sources material from "a vast database of digital books, online writings, and other media."

14 Roughly 25% of the nation's debt was incurred during the Trump Administration. A bipartisan package of legislation, passed toward the end of the Trump administration, added roughly $3.7 trillion to the national debt.

15 Trump's administration agreed to an exchange that freed 5,000 Taliban prisoners. Trump's administration had previously agreed to a prisoner exchange and planned to invite Taliban leaders to Camp David.

16 15 banks in the U.S. had shut down under former President Donald Trump. There were 15 bank failures during Trump's presidency: four in 2020, four in 2019 and seven in 2017.

17 Meta will begin charging a monthly fee for verified accounts. In February, Meta (the parent company of Facebook and Instagram) announced it will begin charging users to have verified accounts.

18 Yes, $725M Facebook Class Action Settlement Is Legitimate. About 250 to 280 million Facebook users are eligible to receive funds from the proposed class action settlement.

News items repeated in the follow-up experiment T3F

1 U.S. President Joe Biden's administration was planning to ban gas stoves over concerns surrounding climate change.

2 The Lee County (Florida) Republican Party passed a proposal seeking to ban the sale and distribution of COVID-19 vaccines in Florida.

3 Federal law does not give U.S. vice presidents authority to declassify government documents.

4 The "AR" in AR-15 stands for assault rifle.

5 Fox News changed its accreditation from "news" to "entertainment."

6 As of January 2023, OpenAI's ChatGPT technology declined to write a poem admiring Donald Trump, but successfully drafted one for Joe Biden.

7 Roughly 25% of the nation's debt was incurred during the previous Trump Administration.

8 A new Facebook rule starts tomorrow that allows the company to use your photos in court and also makes all of your posts and deleted messages public.

9 Will Meta Begin Charging for Verified Accounts?

News claims added to the follow-up experiment T3F

10 California Turned Away Fire Trucks from Oregon Over Smog Certification. (False)

11 Elon Musk Urged People to Stop Donating to Wikipedia. (True)

12 In December 2024, Barron Trump, son of U.S. President Donald Trump, gave up his first-class airplane seat to a military veteran. (False)

13 The United States subsidizes Canada with more than $100 million a year, according to Trump. (False)

14 ABC will pay $15 million to U.S. President Donald Trump to settle a defamation lawsuit involving "This Week" host George Stephanopoulos. (True)

15 As of December 2024, South Carolina lawmakers have proposed a bill that would allow the death penalty for people who get abortions. (True)

16 Jimmy Carter Volunteering Despite Head Injuries at Age 95. (True)

17 AI Models Can Lie and Make Copies To Save Themselves, Researchers Found. (True)

18 Does 'Other' Button on Instagram Show Who's Blocked You? (False)
