# Supplementary material for: The motivation and consequence of fact-checking behavior: An experimental study
Source: PLoS One. 2025 May 23;20(5):e0323105. doi: 10.1371/journal.pone.0323105 (PMC12101777; doi:10.1371/journal.pone.0323105)
Supplement: S3 Appendix — Presents a table of individual characteristics by treatment group. (PDF) [file pone.0323105.s003.pdf]

S3 Appendix. Balance test.

Table S3. Individual characteristics by treatment group.

	T1	T2	T3	T4	Total	Pearson chi^2
**Gender**						
Female	72	73	63	90	298	11.5776
Male	87	85	103	72	347	$p = 0.072$
Non-binary	4	6	3	4	17	
**Age**						
Under 35	62	51	56	58	227	4.9809
35-64	86	96	103	93	378	$p = 0.546$
65 or older	16	18	10	16	60	
**Race**						
American Indian or Alaska Native	2	0	0	3	5	
Asian or Pacific Islander	14	14	10	11	49	
Black or African American	17	17	18	20	72	9.7473
Caucasian	111	119	122	112	464	$p = 0.835$
Hispanic or Latino	10	8	11	9	38	
Mixed racial background	9	7	6	11	33	
**Ideology**						
Conservative	51	52	45	42	190	2.5591
Liberal	105	108	115	120	448	$p = 0.465$
**Party**						
Democrat	84	84	85	90	343	2.5263
Republican	29	34	29	25	117	$p = 0.866$
Independent	46	41	50	48	185	
**Religion**						
Not at all religious	77	76	95	89	337	
Slightly religious	24	28	20	24	96	8.2571
Moderately religious	34	27	28	33	122	$p = 0.508$
Strongly religious	26	32	25	20	103	
**Education**						
High school degree or below	14	24	23	25	86	
Some college but no degree	36	33	31	30	130	10.6149
Associate degree	28	24	17	25	94	$p = 0.562$
Bachelor degree	61	54	73	61	249	
Graduate degree	25	30	25	25	105	
**Income**						
Less than $24,999	33	21	31	28	113	
$25,000 to 49,999	31	44	35	42	152	18.0702
$50,000 to 74,999	31	37	43	40	151	$p = 0.114$
$75,000 to 99,999	24	25	23	32	104	
$100,000 and greater	44	37	29	22	132	
**Exposure to current affairs**						
Hardly at all	9	8	14	11	42	
Only now and then	19	17	22	18	76	6.3986
Some of the time	56	69	59	71	255	$p = 0.699$
Most of the time	79	71	74	66	290	
**Fact checking habit**						
Hardly at all	14	9	22	27	72	
Only now and then	35	24	29	25	113	16.6997
Some of the time	61	65	62	65	253	$p = 0.054$
Most of the time	51	67	56	50	224	
**N**	161	165	169	167	662	
