# Supplementary material for: The motivation and consequence of fact-checking behavior: An experimental study
Source: PLoS One. 2025 May 23;20(5):e0323105. doi: 10.1371/journal.pone.0323105 (PMC12101777; doi:10.1371/journal.pone.0323105)
Supplement: S4 Appendix — Describes a two-way fixed-effects regression analysis of the relationship between accuracy and item-level evaluations. (PDF) [file pone.0323105.s004.pdf]

S4 Appendix. Factor model of accuracy rate.

We can break down subjects' accuracy rate by news items. Figure S4 depicts participants' performance on each news item's evaluation, a pattern that confirms our previous findings about differences across different treatment groups, i.e., paying a bonus does not improve accuracy, whereas the opportunities of receiving fact-checking results do. The figure offers further details about which pieces of news could in particular benefit from fact-checking. The accuracy rates of items 2, 4, 6, 10, 11, 12, 14, 16, and 17 increase substantially in T3 and T4 compared to that in T1 and T2. These items are mainly political news, and their contents are reported in S2 Appendix.

Fig S4. Histogram of accuracy rate by news items.

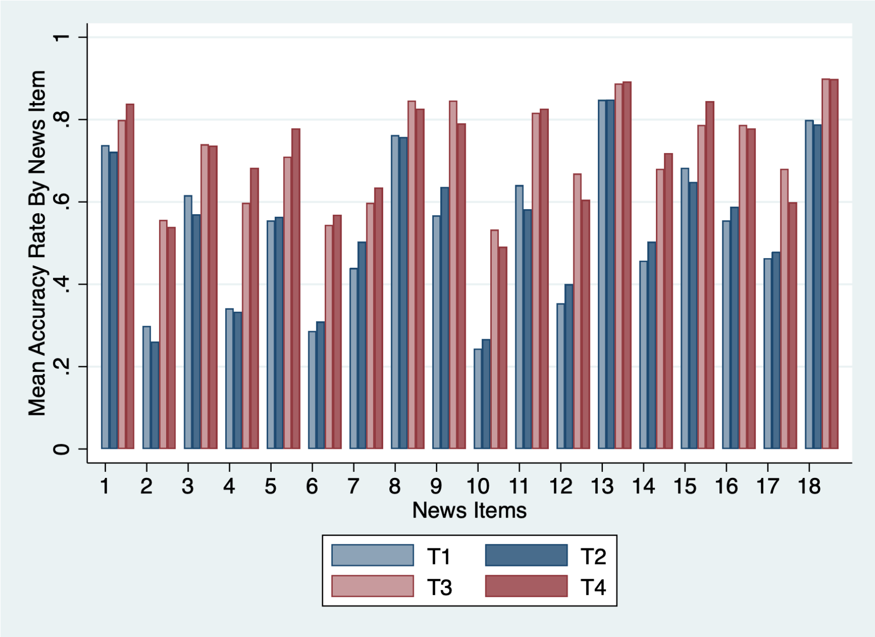

Similarly to our analysis of the determinants of fact-checking, in our examination of the relationship between the accuracy of the participants and their evaluations of the news items' attributes, we estimate the following two-way fixed-effect (TWFE) model at the level of individual-item pairs:

$$Correct_{it} = \alpha_i + \eta_t + \mathbf{X}_{it} \cdot \gamma + \varepsilon_{it} \tag{1}$$

where i is a subject, t is an item, $Correct_{it}$ is an indicator variable that takes the value of 1 if subject i is correct about the authenticity of item t, and takes the value of 0 otherwise. α_i is the individual fixed effect, and η_t is the item fixed effect. $\mathbf{X}_{it}$ is a vector of factors that vary across the individual-item pairs. The standard errors are clustered at the individual level.

Table S4 reports our results. We find that the perceived easiness of evaluating the news item's authenticity has a direct positive effect on accuracy and remains significant across all specifications. The effect is relatively small in magnitude relative to the negative effect easiness has on fact-checking behavior, leading to an overall negative indirect effect on accuracy. Similarly, awareness and willingness to discuss the news with family members

show a slight positive effect, although both are sensitive to the model specification. The lack of a stronger and more consistent effect of awareness on accuracy is likely explained by the individuals overestimating their familiarity with the news items, or by the fact that everyday exposure to news allows individuals to be aware of them without necessarily knowing their authenticity with certainty. Other factors, such as the news item's personal relevance to the respondent, the participant's desire to share the news on social media, and the perceived social importance of the news item, do not significantly affect the likelihood of estimating its authenticity correctly.

Table S4. Determinants of accuracy.

	(1) LPM	(2) LPM	(3) LPM	(4) Logit	(5) Probit
Personal interest	−0.0190	−0.0139	−0.000428	−0.00195	−0.00112
	(0.0107)	(0.0104)	(0.0114)	(0.0582)	(0.0348)
Discuss with family/friends	0.0430***	0.0284*	0.0242	0.122	0.0763*
	(0.0120)	(0.0121)	(0.0123)	(0.0641)	(0.0380)
Repost on social media	0.00409	0.00481	0.0239*	0.115	0.0640
	(0.0111)	(0.0111)	(0.0120)	(0.0627)	(0.0371)
Easiness	0.0351***	0.0274***	0.0211*	0.116*	0.0698*
	(0.00819)	(0.00802)	(0.00959)	(0.0494)	(0.0297)
Awareness	0.0955***	0.0557**	0.0315	0.173*	0.0966
	(0.0162)	(0.0174)	(0.0169)	(0.0874)	(0.0510)
Social importance	−0.0286**	−0.0119	−0.0122	−0.0640	−0.0414
	(0.00886)	(0.00933)	(0.00981)	(0.0503)	(0.0300)
Item fixed effect	No	Yes	Yes	Yes	Yes
Individual fixed effect	No	No	Yes	Yes	Yes
Observations	5922	5922	5922	5796	5796
Adjusted R^2	0.016	0.129	0.184		

Standard errors in parentheses. * $p < 0.05$, ** $p < 0.01$, *** $p < 0.001$
