# Supplementary material for: The motivation and consequence of fact-checking behavior: An experimental study
Source: PLoS One. 2025 May 23;20(5):e0323105. doi: 10.1371/journal.pone.0323105 (PMC12101777; doi:10.1371/journal.pone.0323105)
Supplement: S5 Appendix — Reports item-level average fact-checking rates across treatment groups. (PDF) [file pone.0323105.s005.pdf]

S5 Appendix. Item-wise comparison of fact-checking likelihood.

Table S5. Fact-checking likelihood by item and treatment group.

	Group		
Item	T3	T4	Total
1	0.30	0.28	0.29
	(0.460)	(0.451)	(0.455)
2	0.46	0.42	0.44
	(0.500)	(0.495)	(0.497)
3	0.38	0.33	0.35
	(0.487)	(0.471)	(0.479)
4	0.41	0.49	0.45
	(0.493)	(0.501)	(0.498)
5	0.34	0.37	0.35
	(0.474)	(0.485)	(0.479)
6	0.37	0.39	0.38
	(0.483)	(0.489)	(0.486)
7	0.27	0.23	0.25
	(0.443)	(0.424)	(0.434)
8	0.46	0.39	0.42
	(0.500)	(0.489)	(0.495)
9	0.48	0.51	0.49
	(0.501)	(0.501)	(0.501)
10	0.44	0.38	0.41
	(0.498)	(0.486)	(0.492)
11	0.43	0.38	0.41
	(0.497)	(0.488)	(0.492)
12	0.53	0.43	0.48
	(0.501)	(0.497)	(0.500)
13	0.25	0.28	0.26
	(0.433)	(0.448)	(0.440)
14	0.51	0.51	0.51
	(0.501)	(0.501)	(0.501)
15	0.43	0.50	0.47
	(0.497)	(0.501)	(0.500)
16	0.49	0.50	0.49
	(0.501)	(0.501)	(0.501)
17	0.37	0.31	0.34
	(0.485)	(0.464)	(0.475)
18	0.33	0.27	0.30
	(0.470)	(0.445)	(0.458)
Total	0.40	0.39	0.39
	(0.490)	(0.487)	(0.489)

Standard deviations in parentheses. T1 and T2 are omitted because fact-checking results are not available in those treatment groups.
