# Supplementary material for: The motivation and consequence of fact-checking behavior: An experimental study
Source: PLoS One. 2025 May 23;20(5):e0323105. doi: 10.1371/journal.pone.0323105 (PMC12101777; doi:10.1371/journal.pone.0323105)
Supplement: S6 Appendix — Re-evaluates the main results using an alternative classification of news items. (PDF) [file pone.0323105.s006.pdf]

S6 Appendix. Alternative classification of news items

Our empirical results in Table 5 of the main text are based on the authors' classification of the 18 news items (e.g., Republican slant, Democrat slant, and Neutral slant). To check the sensitivity of the results to alternative categorizations of the news items, we re-do the classification using chatbots powered by advanced Large Language Models (LLMs) and re-run the analysis.

We focus on three different models: ChatGPT-3.5, ChatGPT-4 (OpenAI, USA), and Copilot (formerly Bing AI, Microsoft, USA). While Copilot was built on a foundation of the GPT-4 model, it was designed and further fine-tuned with an emphasis on web search and inclusion of real-time search results into its responses, which can lead to variations in the outcomes compared to GPT-4 (which primarily generates responses based on a pre-existing dataset). The performance of GPT-3.5 and GPT-4 in sentiment analysis, text-based context bias detection, and framing bias identification in media headlines has been shown to be on par with models specifically fine-tuned for such classification and detection tasks [1, 2, 3].

For each model we tested several prompt variations, to check for and ensure consistency in the responses. Upon obtaining results from all three models, we selected three items per category that had the highest assignment confidence, with all three models agreeing on the item's categorization. These selected items were then used for few-shot prompting GPT-4 – a method that is known for enhancing classification accuracy by relying on a limited set of examples, offering an advantage over zero-shot approaches [1]. The LLM classification results are presented in Table S6A.

Table S6A. LLM-based classification of news items.

**News Items/Method**	Authors	Copilot	GPT-3.5	GPT-4	Few-shot prompt GPT-4
1	Rep	Rep	Dem	Neutral	Neutral
2	Rep	Dem	Dem	Neutral	Neutral
3	Rep	Dem	Dem	Dem	Dem
4	Dem	Dem	Neutral	Neutral	Rep
5	Dem	Dem	Neutral	Neutral	Dem
6	Dem	Rep	Neutral	Dem	Dem
7	Neutral	Dem	Neutral	Neutral	Neutral
8	Neutral	Neutral	Neutral	Neutral	Neutral
9	Neutral	Neutral	Neutral	Neutral	Neutral
10	Rep	Rep	Rep	Rep	Rep
11	Rep	Rep	Rep	Rep	Rep
12	Rep	Rep	Rep	Rep	Rep
13	Dem	Dem	Rep	Dem	Dem
14	Dem	Dem	Neutral	Dem	Dem
15	Dem	Dem	Dem	Dem	Dem
16	Neutral	Neutral	Neutral	Rep	Dem
17	Neutral	Neutral	Neutral	Neutral	Neutral
18	Neutral	Neutral	Dem	Neutral	Neutral

Table S6B reports the regression estimates based on the 9 items that were confidently

identified across the LLMs. Republican slants are less likely to be checked. Neither Democrats nor Republicans have a higher propensity to engage in fact-checking than the independents. Interestingly, Republican members are significantly more likely to fact-check the Democratic slants across different model specifications. Consistent with the findings in the main text, this result supports Hypothesis 7 that information incongruence increases fact-checking.

Table S6B. Robustness on classification of news items.

	(1)	(2)	(3)	(4)	(5)
	LPM	LPM	LPM	Logit	Probit
Democrat slant	−0.0278	0.0118	−0.130	−0.599	−0.359
	(0.0219)	(0.0948)	(0.158)	(0.668)	(0.413)
Republican slant	−0.0476*	−0.128	−0.0113	−0.0490	−0.0384
	(0.0217)	(0.0887)	(0.152)	(0.631)	(0.392)
Democratic Party		−0.0529	−0.0332	−0.138	−0.0845
		(0.0369)	(0.0425)	(0.175)	(0.109)
Republican Party		0.0370	−0.0594	−0.243	−0.151
		(0.0583)	(0.0708)	(0.286)	(0.179)
Democrat slant × Democratic Party		0.129**	0.0489	0.203	0.123
		(0.0479)	(0.0578)	(0.240)	(0.148)
Democrat slant × Republican Party		0.0897	0.260**	1.131**	0.693**
		(0.0766)	(0.0969)	(0.417)	(0.256)
Republican slant × Democratic Party		0.0813	0.0789	0.336	0.204
		(0.0493)	(0.0569)	(0.240)	(0.148)
Republican slant × Republican Party		−0.0498	−0.0216	−0.102	−0.0602
		(0.0778)	(0.0958)	(0.395)	(0.245)
News literacy controls	No	Yes	Yes	Yes	Yes
Demographics controls	No	No	Yes	Yes	Yes
Observations	3024	2934	2790	2790	2790

Standard errors in parentheses. * $p < 0.05$, ** $p < 0.01$, *** $p < 0.001$

References

1. Pastorino V, Sivakumar JA, Moosavi NS. Decoding news narratives: A critical analysis of large language models in framing bias detection; 2024.

2. Rathje S, Mirea DM, Sucholutsky I, Marjieh R, Robertson C, Van Bavel JJ. GPT is an effective tool for multilingual psychological text analysis; 2023. Available from: `osf.io/preprints/psyarxiv/sekf5`.

3. Wen Z, Younes R. ChatGPT v.s. media bias: A comparative study of GPT-3.5 and fine-tuned language models. Applied and Computational Engineering. 2023;21(1):249–257. doi:10.54254/2755-2721/21/20231153.
