# Supplementary material for: The motivation and consequence of fact-checking behavior: An experimental study
Source: PLoS One. 2025 May 23;20(5):e0323105. doi: 10.1371/journal.pone.0323105 (PMC12101777; doi:10.1371/journal.pone.0323105)

## S7 Appendix. Additional treatment of large bonus.

In our main study, we find that monetary incentives do not show any significant effect on fact-checking behavior (Result 4), thereby contradicting the VoI theory. One possible concern is that the bonus in our original experiment was too small to meaningfully influence behavior. To address this issue and to validate our results we conducted a follow-up study referred to as T4L throughout this section. This study closely followed the procedures and parameters set for the T4 treatment group: participants have 9 opportunities to receive a credible report about the news item under consideration and they receive a monetary bonus for each accurate evaluation of news authenticity. The main feature of the new treatment is the significant bonus increase - while previously we rewarded each correct estimation with \$0.2, for the T4L treatment group we have increased the bonus payment to \$1 per correct estimation.

Figure S7A plots the 95% confidence interval of the mean fact-checking likelihood aggregated over all of the rounds for treatments T3 (fact-check available, no monetary bonus), T4 (fact-check available, \$0.2 bonus), and T4L (fact-check available, \$1 bonus). The mean fact-checking rates show no significant differences between the three groups. The distributions of fact-checking likelihood across both of the monetary incentive treatments, T4 and T4L, show no significant difference, as confirmed by the Mann-Whitney non-parametric test ($p = 0.066$). Therefore, increasing the bonus from \$0.2 to \$1 had no significant effect on the fact-checking behavior.

Additionally, our results show that there was no significant improvement in the accuracy rate despite the higher bonus payment ($p$=0.98). Figures S7B and S7C confirm that result visually, alleviating our concerns that the initially smaller accuracy reward was the reason for the lack of effort. Taken together, our results indicate that even considerably increased monetary incentives do not lead to more frequent fact-checking behavior or more careful evaluation of information, which affirms the result found in the main study.

**Fig S7A. Mean fact-checking rate by treatment group.**

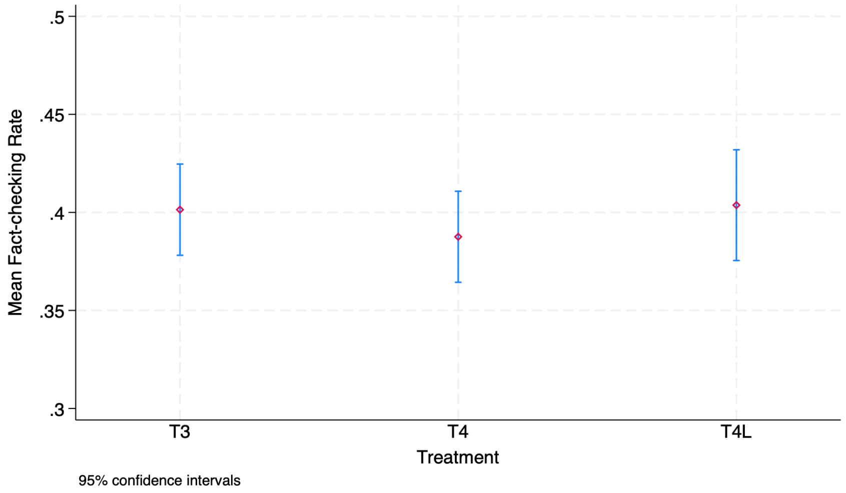

**Fig S7B. Box plots of individual accuracy rate by treatment group.**

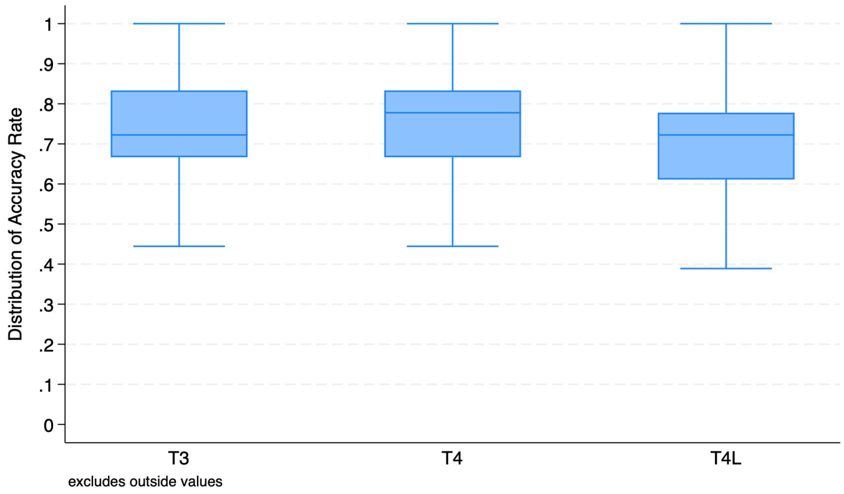


**Fig S7C. Mean accuracy rate by treatment group.**

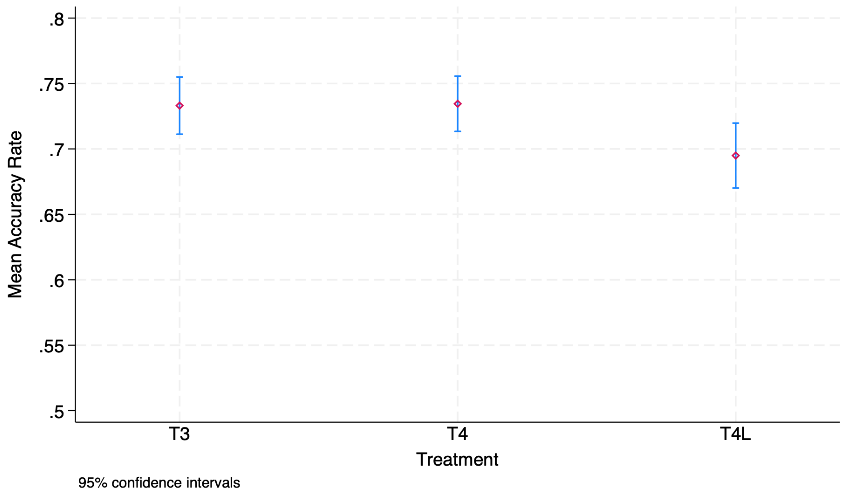

Supplement: S7 Appendix — Reports results from a follow-up experiment with a significantly larger bonus for each accurate judgment. (PDF) [file pone.0323105.s007.pdf]
